# Dual-Band 802.11 RF Energy Harvesting Optimization for IoT Devices with Improved Patch Antenna Design and Impedance Matching

**DOI:** 10.3390/s25041055

**Published:** 2025-02-10

**Authors:** Ashraf Ali, Rama Eid, Digham Emad Manaseer, Hussein Khaled AbuJaber, Andrew Ware

**Affiliations:** 1 Department of Electrical Engineering, Faculty of Engineering, The Hashemite University, Zarqa 13133, Jordan; 2 Faculty of Computing, Engineering and Sciences, University of South Wales, Pontypridd CF37 1DL, UK

**Keywords:** Wi-Fi energy harvesting, Internet of Things (IoT), dual-band antenna, microstrip antenna, impedance matching, reflection coefficient

## Abstract

This paper investigates the feasibility of harvesting Radio Frequency (RF) energy from the Wi-Fi frequency band to power low-power Internet-of-Things (IoT) devices. With the increasing prevalence of IoT applications and wireless sensor networks (WSNs), there is a critical need for sustainable energy sources that can extend the operational lifespan of these devices, particularly in remote locations, where access to reliable power supplies is limited. The paper describes the design, simulation, and fabrication of a dual-band antenna capable of operating at 2.4 GHz and 5 GHz, the frequencies used by Wi-Fi. The simulation and experimental results show that the proposed design is efficient based on the reflection coefficient. Using a high-frequency simulator, we developed two C-shaped and an F-shaped microstrip antenna design, optimized for impedance matching and efficient RF–DC conversion.The captured RF energy is converted into usable electrical power that can be directly utilized by low-power IoT devices or stored in batteries for later use. The paper introduces an efficient design for dual-band antennas to maximize the reception of Wi-Fi signals. It also explains the construction of an impedance-matching network to reduce signal reflection and improve power transfer efficiency. The results indicate that the proposed antennas can effectively harvest Wi-Fi energy, providing a sustainable power source for IoT devices. The practical implementation of this system offers a promising solution to the energy supply challenges faced by remote and low-power IoT applications, paving the way for more efficient and longer-lasting wireless sensor networks.

## 1. Introduction

The rapid evolution of Internet of Things (IoT) applications and wireless sensor networks (WSNs) has highlighted the critical need for efficient energy conservation methods for low-power devices. A significant challenge these devices face, especially when deployed in remote locations, is their reliance on traditional batteries with fixed power ratings and limited lifespans. Traditional energy supply methods hinder the full potential of IoT applications due to the frequent need for battery replacements or recharging. Therefore, there is a growing necessity to explore alternative energy sources that can provide a sustainable and continuous power supply to extend the operational lifetime of these devices. Energy harvesting, which involves capturing and converting ambient energy into usable electrical energy, is emerging as a promising solution to power such wireless devices. Energy harvesting involves capturing and converting ambient energy from the environment into usable electrical energy. Among the various ambient energy sources, Radio Frequency (RF) energy, specifically from Wi-Fi signals, is highly attractive due to the ubiquitous presence of Wi-Fi in modern environments. The basic structure of a Wi-Fi energy-harvesting system comprises a receiving antenna, an impedance-matching circuit, and an RF-DC rectifier, which collectively capture and convert RF energy into electrical power.

Implementing an effective Wi-Fi energy-harvesting system requires a careful design process, which includes the following critical components: Designing an efficient dual-band antenna to enhance the reception of Wi-Fi signals. Constructing an impedance-matching network to minimize signal reflection and maximize power transmission and reception efficiency. A rectifying circuit was developed to convert AC signals captured by the antenna into a DC power, suitable for immediate use or storage in energy storage elements. The S11 parameter measures the reflection coefficient. It indicates the efficiency of the impedance matching between the antenna and the energy-harvesting circuit. A lower S11 value means better impedance matching producing more RF power from the Wi-Fi signal. For instance, a typical Wi-Fi antenna designed for energy harvesting will aim for an S11 value below −10 dB [1], corresponding to absorbing approximately 90 % of the incident power. An S11 < −20 dB is preferred for higher efficiency, meaning 99% of the power is absorbed [2].

The primary aim of this paper is to explore the feasibility of harvesting RF energy to power low-power IoT devices by utilizing the Wi-Fi frequency band. The research’s objectives are multi-faceted, encompassing the design, simulation, fabrication, and testing of an antenna with an impedance-matched circuit. The research aims to create a sustainable power source for IoT devices, reducing their dependence on traditional batteries and extending their operational lifespan. Energy harvesting has emerged as a crucial technology for powering low-power devices, especially in the Internet of Things (IoT) and Wireless Sensor Networks (WSNs). The ability to convert ambient energy from the environment into usable electrical power provides a sustainable alternative to traditional battery-powered systems, which suffer from limited lifespans and frequent maintenance requirements. Among various energy harvesting methods, Radio Frequency (RF) energy harvesting, particularly from Wi-Fi signals, stands out due to the ubiquitous presence of Wi-Fi in modern environments. As previously mentioned, Wi-Fi energy harvesting relies on capturing ambient RF energy from Wi-Fi signals, typically emitted in the 2.4 GHz and 5 GHz frequency bands. The power density of Wi-Fi signals is relatively low, ranging from $10^{-3}$ to $10^{-4}$ watts per square meter at typical Wi-Fi transmission distances, translating to energy harvesting potentials of 10–100 μW [1,3]. These figures are lower compared to solar and vibration-based energy harvesting systems. Solar energy and vibration-based energy harvesting are more power-dense than Wi-Fi energy harvesting. Solar energy can generate up to 200–300 ${W/m}^{2}$ under sunlight, while vibration harvesting systems can generate 0.1–1 mW from mechanical vibrations [4,5]. However, these technologies are location-dependent in that solar energy requires exposure to sunlight, and vibration energy requires consistent mechanical motion. In contrast, Wi-Fi energy harvesting can operate continuously if a wireless network exists. The availability of Wi-Fi signals and the strength of the signals depend heavily on the local infrastructure, network density, and regulatory policies, which can vary significantly across different countries.

The strength of the signal is in accordance with the local infrastructure, network density and bandwidth availability, and regulatory framework. Various countries have adopted different regulatory standards for Wi-Fi frequency bands and bandwidth availability, as well as the impact of signal strength, interference levels, and data transfer rates. Wi-Fi operates in the United States and EU and has relatively high spectrum availability.

The U.S. Federal Communications Commission (FCC) allows for the unlicensed use of these bands, contributing to a broader deployment of Wi-Fi networks. These regions tend to have dense Wi-Fi infrastructure, which can provide higher energy harvesting potential from ubiquitous Wi-Fi signals in urban areas [6]. China has more stringent regulations over spectrum usage. In rural areas or regions with a less robust Wi-Fi infrastructure, the Wi-Fi signal strength may be weaker, reducing the energy harvesting efficiency [7,8,9].

In urban centers, however, the high density of Wi-Fi routers and public hotspots can increase the available energy for harvesting. Finally, Japan offers one of the highest bandwidth availabilities for Wi-Fi signals, with a relatively dense network of Wi-Fi access points. The 5 GHz band is widely utilized, offering a greater bandwidth and less interference, which could allow for a higher energy harvesting efficiency [7]. Furthermore, Japan’s extensive urban wireless infrastructure contributes to higher signal strength, making it more suitable for energy harvesting systems.

Ref. [10] details the fundamental principles of RF energy harvesting, including the importance of efficient antenna design and impedance matching in maximizing the power transfer efficiency.

Antenna design plays a pivotal role in RF energy-harvesting systems. The efficiency of an antenna in capturing RF energy directly impacts the system’s overall performance. Various designs, including microstrip antennas, have been investigated for their suitability in energy-harvesting applications. For instance, in [11], the authors demonstrated a dual-band antenna operating at 2.4 GHz and 5 GHz for harvesting energy from Wi-Fi signals. Their study emphasized optimizing antenna dimensions and materials to achieve high efficiency.

In the context of this research, dual-band antennas with C-shaped and F-shaped designs were developed and simulated using a high-frequency simulator. As demonstrated in previous research [12], the choice of these designs was influenced by their ability to operate efficiently at the specified Wi-Fi frequencies. Impedance matching is critical in minimizing signal reflection and maximizing power transfer from the antenna to the rectifying circuit. The use of Smith Charts for designing impedance-matching networks has been extensively documented in the literature.

In other research, the authors illustrated the effectiveness of using Smith Charts to optimize impedance-matching networks for RF energy harvesting systems [13]. Rectifying circuits, which convert the captured AC signals into DC power, are another essential component of RF energy-harvesting systems. In [14], various rectifier topologies and their performance in different frequency bands were explored. Their findings underscore the importance of selecting appropriate rectifier components to achieve a high conversion efficiency. The practical applications of RF energy harvesting are vast, ranging from powering IoT devices to wearable technologies and remote monitoring systems. In [15], the researchers discussed the potential of RF energy harvesting in extending the battery life of portable devices and enabling completely wireless sensor networks. Their study highlighted the sustainability benefits and operational efficiencies of deploying RF energy-harvesting systems in real-world scenarios. Despite the promising potential of RF energy harvesting, several challenges remain. These include the variability of ambient RF energy sources, energy conversion efficiency, and integration of energy storage solutions. Future research will address these challenges through advanced materials, innovative antenna designs, and improved energy management systems [16].

This paper is structured to provide a comprehensive overview of the development and implementation process of the RF energy-harvesting system. Subsequent sections delve into the specifics of antenna design, including the design specifications and models of various antennas considered for this research. Section 2 presents some related studies on antenna designs and impedance-matching circuits. Section 3 focuses on the design process of the antenna, detailing the geometric modelling, design specifications, and associated results and plots from the antenna simulations, highlighting the performance of the selected and unselected antenna designs based on criteria such as S11 parameters, VSWR, and gain. Section 4 discusses the construction of the impedance-matching network using Smith Charts, and Section 5 ends with the conclusions.

## 2. Models of Various Antennas and Related Work

**Figure 1:**
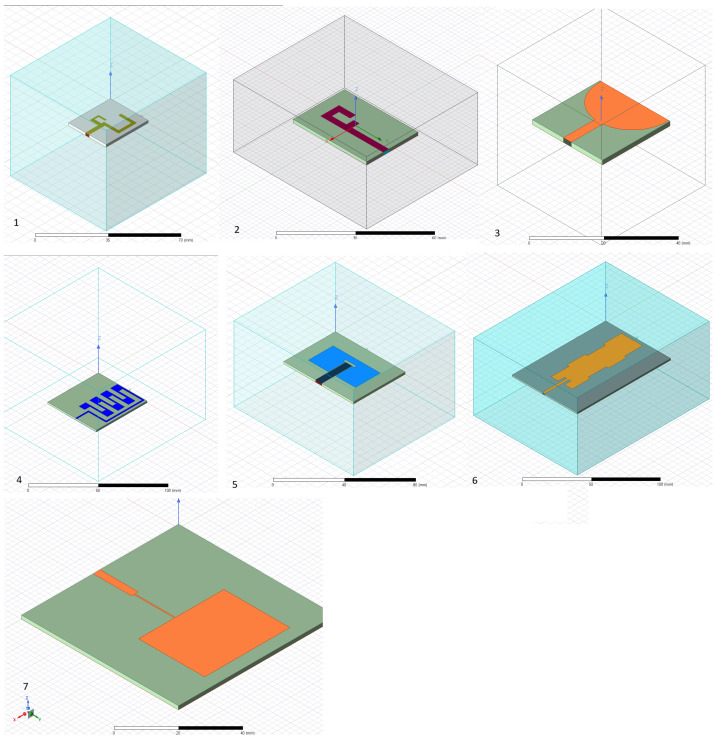
(**1**) Two C-shaped antennas. (**2**) F-shaped antenna. (**3**) Ultra-wideband antenna. (**4**) Zig-zag multiband antenna. (**5**) Dual-band microstrip antenna. (**6**) Dual-band antenna. (**7**) Regular patch antenna. (The gray shade in all subfigures represents the layer of non-copper material, while all other colors represent the layer of copper conductors for the top layer).

This section details the designs of multiple antennas simulated using a finite state element simulator, as shown in Figure 1. A performance evaluation for each design follows in the next section. HFSS, as a finite-state simulator, can provide measurements crucial for understanding the performance of the antenna design. Here is a brief introduction to the types of results that can be expected from an HFSS simulation for an antenna:

- S-parameters (Scattering Parameters): These are the most common results obtained from HFSS simulations. They indicate how an antenna radiates and receives energy. For example, S11, or the return loss, shows how much power is reflected into the source, helping to minimize efficient radiation.
- Radiation patterns: HFSS can generate 3D plots showing how an antenna radiates energy into space. This is essential for understanding the antenna’s directivity and gain.
- Field distributions: HFSS provides electric and magnetic field distributions around the antenna. This helps visualize the near-field and far-field characteristics and ensures compliance with safety standards for electromagnetic exposure.
- Impedance: The input impedance of the antenna is another critical result. It must be matched to the transmission line for maximum power transfer.
- VSWR (Voltage Standing Wave Ratio): this is related to the S-parameters and indicates how well the antenna is impedance-matched to the transmission line.
- Current distribution: HFSS can show the current distribution on the antenna’s surface, which helps identify areas of high radiation or potential losses.

Recent research into RF energy harvesting for IoT devices highlights some promising solutions for efficiently converting ambient RF signals into power, which is essential for sustaining low-power devices like sensors and medical implants. A study by [17] shows that matching the impedance between components is key to maximizing the energy transfer efficiency. Using a Cockcroft–Walton voltage multiplier, they enhance the power output of circuits designed for IoT applications by addressing mismatches that usually reduce energy capture [17].

The F-shaped and C-shaped antennas hold notable value in wireless energy harvesting, especially for applications focused on optimizing ambient energy capture for low-power devices. Both designs enhance energy efficiency in different ways, tailored to specific operational needs.

The C-shaped antenna, or C-slot circular polarized antenna, operates effectively within the 2.3–2.4 GHz frequency range. It employs dual-feed configurations that enable both Left-Hand and Right-Hand Circular Polarization (LHCP and RHCP), making it ideal for applications that benefit from circular polarization. This design integrates a high-frequency rectifier circuit with a thin-film solar cell, resulting in a compact, self-sustaining power source suited for small electronic devices [18].

In contrast, the F-shaped antenna, and particularly the inverted-F antenna (IFA), supports triple-band functionality, covering the EGSM-900, GSM-1800, and LTE-2600 bands, This makes it a versatile option for RF energy harvesting across multiple frequency bands. Advanced optimization techniques further enhance the F-shaped antenna’s performance, allowing it to maintain a high energy capture efficiency across varied environments. This is especially useful in applications requiring adaptability to different RF sources [19].

Ultimately, both the F-shaped and C-shaped antennas demonstrate substantial energy harvesting potential, though the choice between them depends on application-specific requirements. The C-shaped design is ideal for circular polarization needs, while the F-shape provides broader frequency adaptability, offering flexibility for diverse RF harvesting applications.

For healthcare, ref. [20] developed an advanced rectenna, type of antenna designed specifically for energy harvesting, operating in the ISM band at 2.45 GHz. This design achieved a 72 % conversion efficiency, making it well suited for powering low-power medical devices continuously and reducing reliance on batteries. In a related study, the team designed another high-efficiency rectenna that provides a stable energy source to IoT healthcare devices, demonstrating the technology’s potential to improve reliability in applications where consistent power is essential [21].

A separate line of research focuses on making energy harvesters highly sensitive to very low input power, as seen in a study on ultra-low-power converters for wearable IoT devices. This technology leverages sensitive RF-to-DC converters that operate efficiently even when RF power is minimal, supporting devices like health monitors and other IoT wearables that need continuous low-power operation [22].

## 3. Design

Designing antennas for energy-harvesting systems involves a careful balance to ensure the antenna works efficiently while fitting into the available space and conditions. One of the key challenges is figuring out the right size and shape for the antenna, so it can capture as much ambient energy as possible. The antenna must also be tuned to pick up specific frequencies from energy sources like radio waves or electromagnetic fields, which can vary depending on where the system is used. In many cases, trade-offs exist between how focused or broad the antenna’s signal reception should be and how much energy it can capture. This section will present different antenna designs, showing the performance metrics for each one.

### 3.1. Two C-Shape Antenna

A two C-shaped antenna design for energy harvesting must balance size, material, and performance to capture ambient energy effectively. Its compact, flexible shape allows it to fit into small spaces but tuning it to resonate at the right frequency for maximum energy capture is key. Materials with high conductivity are needed to minimize losses, and the broad radiation pattern of the C-shaped design helps capture energy from multiple directions. Environmental factors like temperature and interference must also be considered to maintain performance in real-world conditions. Overall, the two C-shaped antennas offer a space-efficient solution for energy harvesting but require the careful optimization of their structure and integration with the power circuitry. More details about the proposed antenna design are shown in the next section.

#### 3.1.1. Antenna Structure

**Figure 2:**
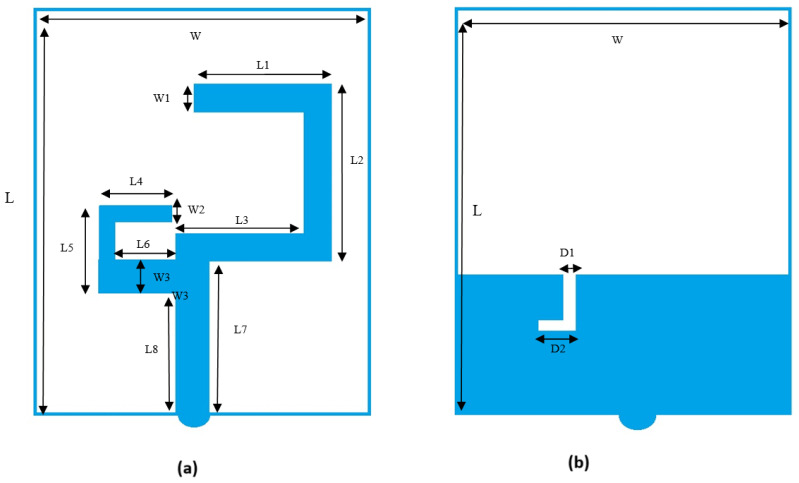
Geometry of proposed antenna: (**a**) Top view. (**b**) Bottom view.

The primary purpose is to design a compact, easy-to-manufacture, cheap, and high-performance microstrip patch antenna. The antenna is printed on the substrate of FR4 with two C-shaped strips, as shown in Figure 2a, and a microstrip feed line on the front side. There is an L slot in the ground plane on the bottom side, as shown in Figure 2b. The overall geometric size of the antenna is 29 × 26 × 1.6 mm^3^. The antenna bandwidth is 140 MHz and 552 MHz at 2.45 GHz and 5 GHz, respectively.

**Figure 3:**
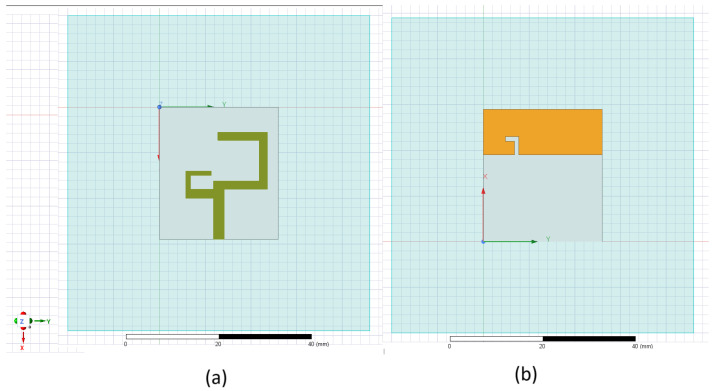
(**a**) Top view. (**b**) Bottom view (The gray shade in all subfigures represents the layer of non-copper material, while all other colors represent the layer of copper conductors for the top layer).

Table 1 summarizes the antenna’s physical dimensions defined in Figure 2. The exact size of the board, location, and dimensions of traces define the precise performance of the entire antenna. This will be identified in different scenarios, as explained in the next section. Figure 3a,b show the design.

#### 3.1.2. Antenna Performance and Discussion

Two C-shaped antennas were designed with two variations. The L slot in the ground plane dimension parameters (D1 and D2) has been altered to be 1 mm and 4 mm, respectively, which will be referred to as the first assumption design in later sections. The other variation is changing (D1 and D2) the L slot to be 1 mm and 3 mm, respectively; this will be referred to as the second assumption design in later text.

**Figure 4:**
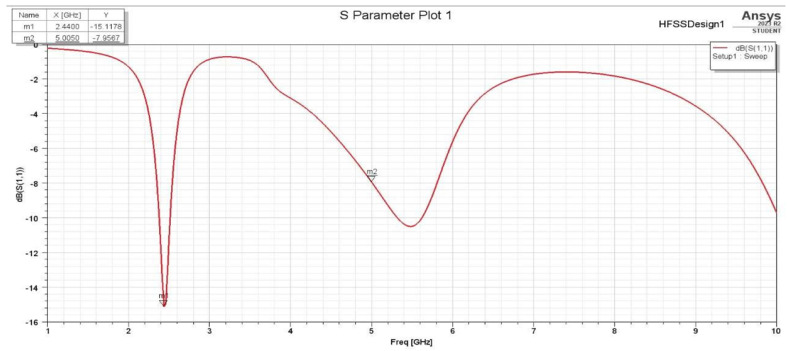
S11 for the first assumption.

**Figure 5:**
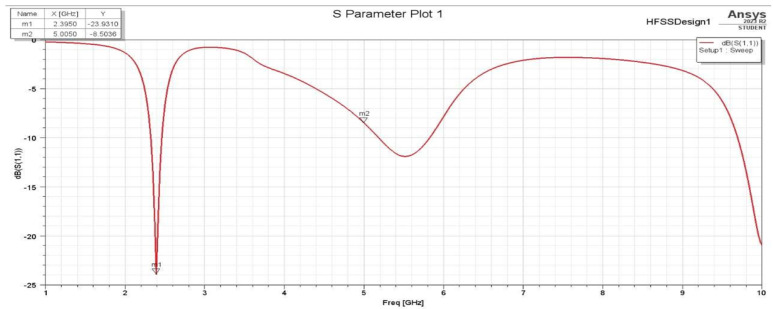
S11 for the second assumption.

It has been noticed that these two changes in the L slot are acceptable in terms of the achieved performance. On the other hand, it was found that the changing position of the L slot on the Y-axis and the length of the L slot along the X-axis affect the S11 plots and gain. The S11 for the second assumption is more selective for the aim frequencies. Also, the frequencies have slightly shifted to the lower-frequency side, which indicates the required frequencies (2.4 GHz and 5 GHz). With a frequency sweep from 1 GHz to 10 GHz, it is clear that the antenna S11 parameter shown in Figure 4 is more than that shown in Figure 5, with a wider bandwidth for both frequencies, 2.4 GHz and 5 GHz.

**Figure 6:**
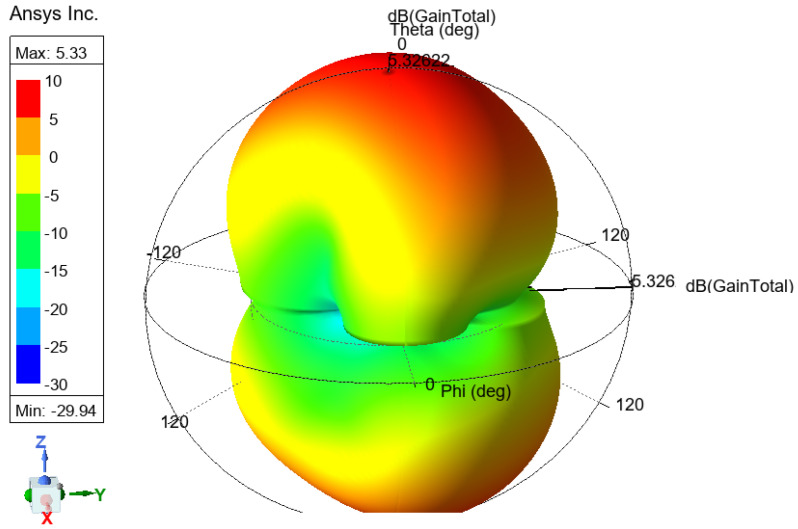
Three-dimensional gain plot for the first assumption.

**Figure 7:**
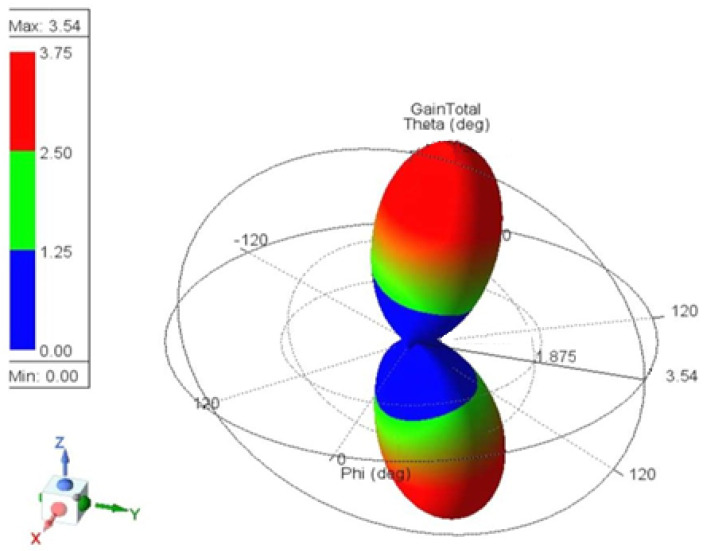
Three-dimensional gain plot for the second assumption.

The antenna gain measures how effectively an antenna converts input power into radiated power in a specific direction and represents it differently. Figure 6 and Figure 7 show the 3D gain plot for the mentioned antennas.

**Figure 8:**
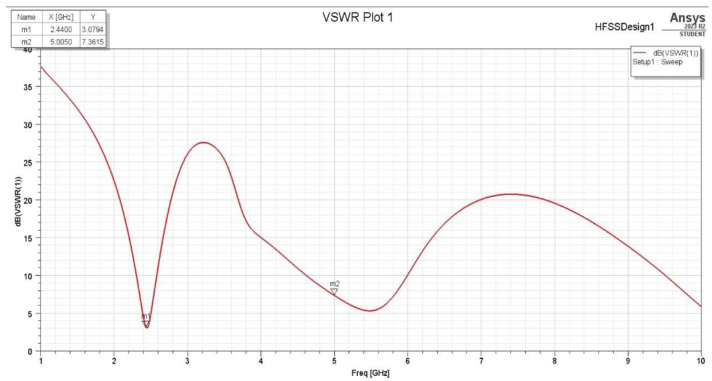
VSWR for first assumption.

**Figure 9:**
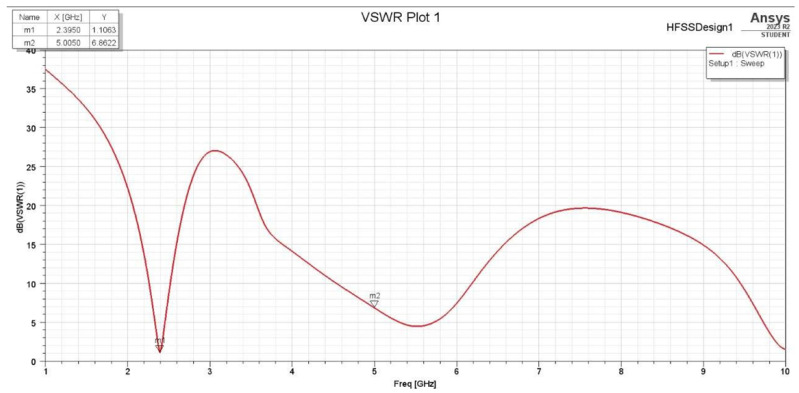
VSWR for the second assumption.

The VSWR (Voltage Standing Wave Ratio) measures how well an antenna matches the transmission line. It indicates the power transfer efficiency between the antenna and the transmission line. Figure 8 and Figure 9 show the VSWR measured in dB for the first and second assumptions. The reflection coefficient, commonly denoted by the Greek letter gamma Γ, represents the ratio between the reflected and incident waves at a specific reference plane. This value varies from −1 (for a short-circuited load) to +1 (for an open-circuited load) and is zero when the load is impedance-matched. The reflection coefficient Γ can be calculated from the complex load impedance $Z_{l}$ and the characteristic impedance of the transmission line $Z_{0}$, which may also be complex:

$$\Gamma =\frac{Z_{l}-Z_{0}}{Z_{l}+Z_{0}}$$

The magnitude of Γ, |Γ| represents the power of the reflected wave when squared, which recalls a historical connection to voltage waveforms.

Now, we can define the Voltage Standing Wave Ratio (VSWR), also called the Standing Wave Ratio (SWR), as a scalar value:

$$\mathrm{VSWR}=\frac{1+|\Gamma |}{1-|\Gamma |}$$

Alternatively, in terms of *S*-parameters, the VSWR can be expressed as

$$\mathrm{VSWR}=\frac{1+|S_{11}|}{1-|S_{11}|}$$

### 3.2. F-Shaped Dual Band

#### 3.2.1. Antenna Structure

**Figure 10:**
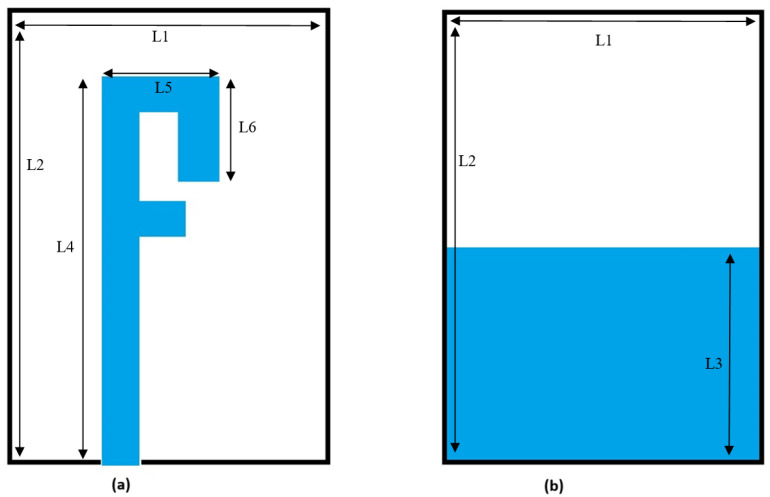
Geometry of proposed antenna: (**a**) Top view. (**b**) Bottom view.

The antenna is designed and optimized to capture energy from the environment at a Radio Frequency range of Wi-Fi bands 2.4 GHz and 5 GHz. The obstacle lies in creating the antenna for both the 2.4 and 5 GHz bands. Figure 10 shows the geometry of the proposed antenna. The F-shaped monopole antenna is designed with an FR4 substrate having a permittivity of 4.4 and a width of 1.6 mm. The design consists of an F-shaped patch and a limited ground plane. The design parameters of the antenna are illustrated in Table 2.

#### 3.2.2. Antenna Performance and Discussion

**Figure 11:**
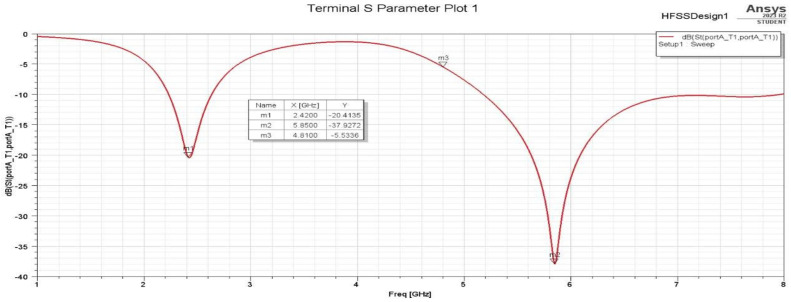
F-shaped antenna S11 plot.

The effectiveness of the chosen antenna can be determined by several factors, including how well it reflects the signal to the transmitter (return loss), how much of the signal is concentrated in a particular direction (gain), and the standing wave ratio (VSWR). Here, to visualize the dips in the S11 dB plot, the frequency sweep was configured from 1 GHz to 8 GHz. As is evident in Figure 11, the antenna appears to be well matched at frequencies 2.42 GHz and 5.85 GHz, with exceptionally minimal signal reflection due to its low return loss.

**Figure 12:**
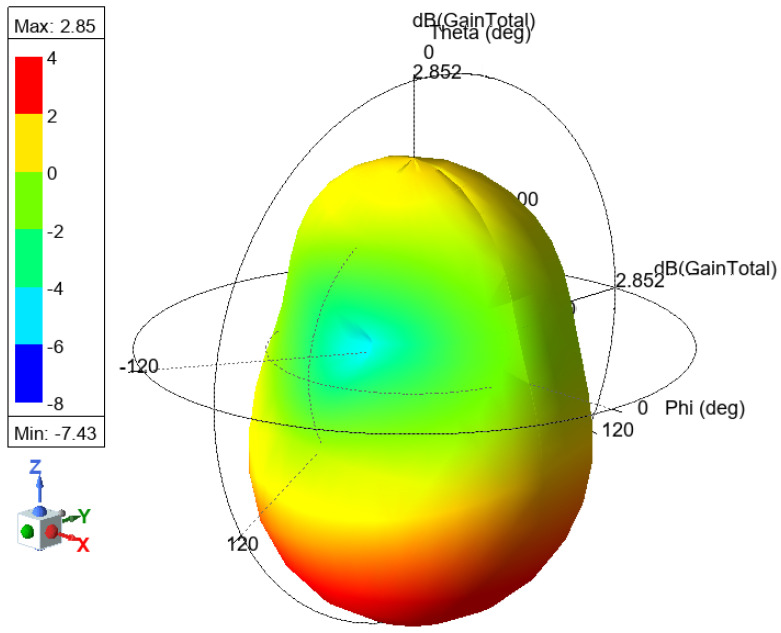
F-shaped antenna 3D gain plot.

The antenna gain measures how effectively an antenna converts input power into radiated power in a specific direction and represents it in a different direction. Figure 12 shows the 3D gain plot for the mentioned antenna.

**Figure 13:**
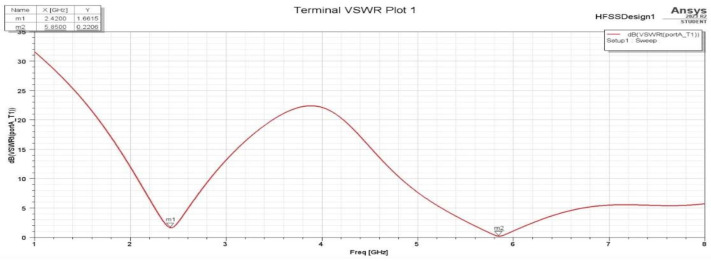
VSWR plot.

The VSWR vs. frequency graph shown in Figure 13 reveals resonances at two distinct frequencies: 2.42 GHz and 5.85 GHz. The VSWR reaches its lowest values at these frequencies, respectively: 1.66 and 0.22.

A summary of the three antenna designs presented is shown in Table 3. The variations between the 2.4 GHz and 5 GHz bands in terms of the S11 parameter emerges since the 5 GHz band operates at a higher frequency, meaning it is more sensitive to antenna design and impedance matching. Achieving a low S11 value is more challenging due to the narrower bandwidth and the higher frequency. Slight mismatches between the antenna and the transmission line can result in higher reflection and less efficient energy harvesting. For the 5 GHz band, it is still essential to achieve S11 < −10 dB [1]. Because of the higher frequency and narrower bandwidth, the antenna’s impedance must be more precisely matched, making it more challenging to achieve an optimal S11 parameter. However, compared to similar works, the antenna design presented in this research outperforms the ones found in the literature, especially for the 5 GHz band, as shown in Table 4.

**Figure 14:**
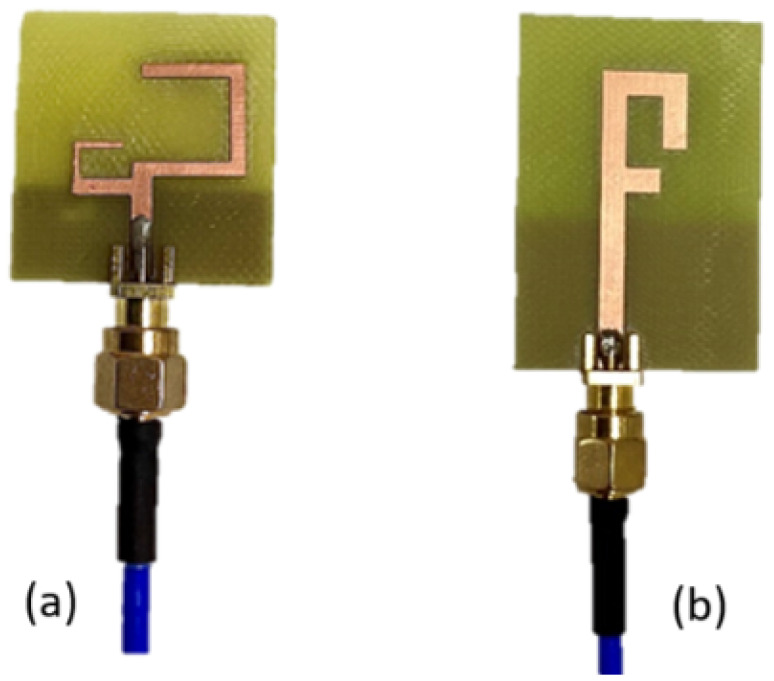
(**a**) Fabricated two-C-shaped antenna. (**b**) Fabricated F-shaped antenna.

The two antennas, a C-shaped and an F-shaped antenna, have been fabricated using a CNC PCB milling machine. The fabrication process involved the precise machining of the antenna structures to ensure accurate dimensions and optimal performance; an SMA was then soldered to the feed line. The fabricated antennas are shown Figure 14.

**Figure 15:**
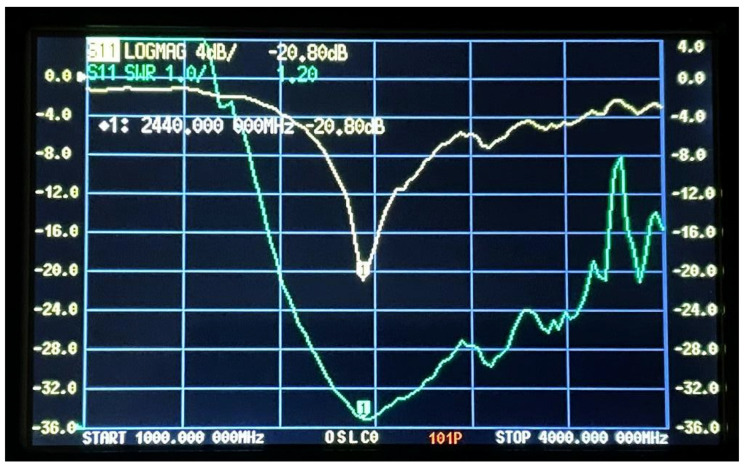
VNA experimentalS11 and VSWR for the two C-shaped antenna.

**Figure 16:**
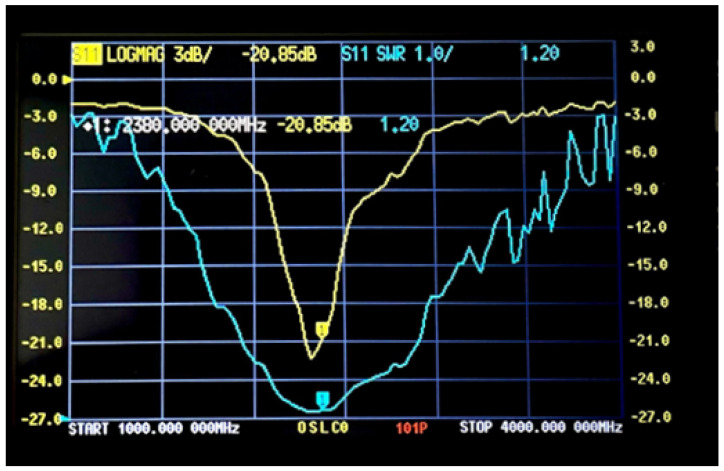
VNA experimental S11 and VSWR for the F-shaped antenna.

The S11 parameters and VSWR results were obtained experimentally after connecting the fabricated antennas to a Vector Network Analyzer (VNA). Figure 15 and Figure 16 show the VSWR and S11 plots for the two C-shaped and F-shaped antennas, respectively. The S11 coefficient for the F-shaped and two C-shaped antennas is −20.8 dB at 2.44 GHz and −20.85 dB at 2.38 GHz, respectively. Similarly, the VSWR was measured as 1.2 for both antennas. Finally, Table 5 compares between the experimental and simulated results for the VSWR and S11 in both antennas. The practical results align closely with the simulation results.

The S11 parameter plays a key role in determining the energy harvesting efficiency from Wi-Fi signals. While the S11 parameter itself does not directly give the net DC power that can be stored, it influences the amount of RF power that can be harvested by an antenna, affecting how much energy can be stored in a battery or supercapacitor. The net DC power that can be stored depends not only on the S11 parameter but also on the efficiency of the energy-harvesting system, which includes the rectifier circuit and the DC–DC converter. The rectification efficiency typically ranges from 50% to 80%, depending on the technology used, while the DC–DC conversion efficiency usually falls between 80% and 90% [27].

This means that the overall system efficiency, or the total energy conversion efficiency $\eta_{\mathrm{total}}$, typically ranges from 40% to 72% when both stages are accounted for.

The power received by the RF frontend can be calculated as

$$P_{\mathrm{RF}}=P_{\mathrm{incident}}\left(1-\right|S_{11}{|}^{2})$$

For example, an incoming RF signal of −20dBm (about 0.01mW) with $S_{11}<-20\;\mathrm{dB}$ would result in 99% absorption. This means the following:

$$P_{\mathrm{RF}}=0.01\;\mathrm{mW}\times 0.99=0.0099\;\mathrm{mW}$$

Values of $S_{11}$ such as −10dB and −20dB would result in the antenna harvesting 90% and 99% of the incident power, respectively.

The DC converter efficiency is typically 70–80% for rectifiers and 80–90% for DC–DC converters. Considering the total efficiency $\eta_{\mathrm{total}}=0.595$, the DC power can be calculated as

$$P_{\mathrm{DC}}=P_{\mathrm{RF}}\times \eta_{\mathrm{total}}=0.0099\;\mathrm{mW}\times 0.595=0.0059\;\mathrm{mW}$$

This means approximately 0.0059mW of the net DC power could be stored.

## 4. Impedance Matching

When using HFSS, the Smith Chart tool can be integrated to facilitate the impedance-matching process. This involves creating a matching network that transforms the antenna’s impedance to the desired value, typically 50 ohms. By linking the Smith Chart tool with the creation of an impedance-matching network, we can efficiently optimize their antenna designs for better performance and integration into RF systems. Smith Charts plot the antenna impedance versus operating frequencies, providing a visual reference for solving impedance mismatches. The lines across the chart are based on multiple equations, illustrating reflection coefficients across various impedance levels.

When designing impedance-matching networks, we aim to present conjugate-matched impedances to the source and load. This means that the real parts of the impedances (resistances) should match, and the imaginary parts (reactance) should be equal in magnitude but opposite in sign. The Smith Chart has become an important avenue for comparing and characterizing the performance of microwave circuits.

**Figure 17:**
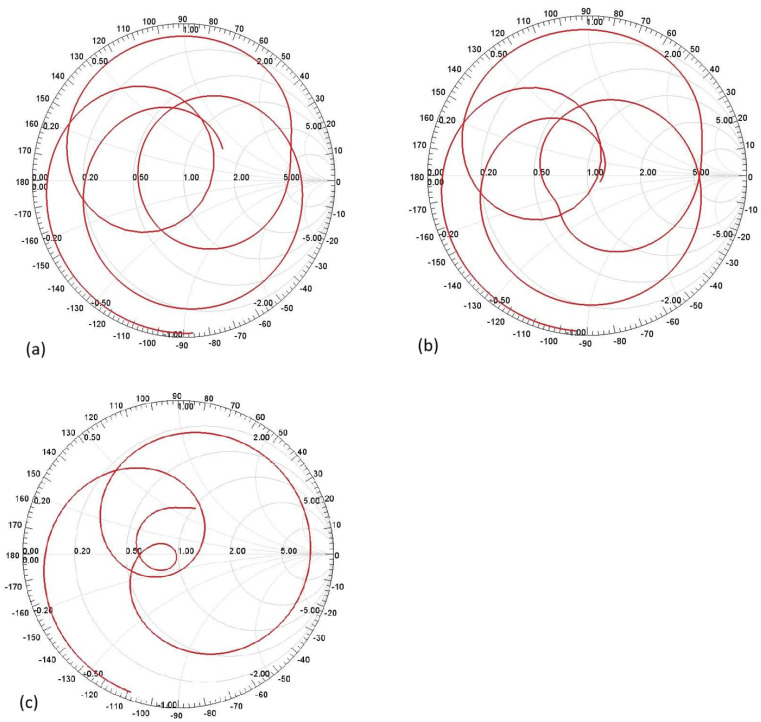
(**a**) Smith Chart of first assumption of two-C-shape antenna. (**b**) Smith Chart of second assumption of two-C-shape antenna. (**c**) Smith Chart of F-shaped antenna.

Figure 17a shows the Smith Chart for the first assumption of two C-shaped antennas. Similarly, Figure 17b shows the Smith Chart for the second assumption of the two C-shaped antennas, and Figure 17c shows the Smith Chart for the F-shaped antenna.

Implementing a circuit using the Smith tool required an iterative design procedure. The chart shows how the current impedance is tuned compared to the desired ideal match by adding series or parallel combinations of inductors and capacitors to deviate the equivalent impedance towards the center of the chart (the matched condition).

After integrating the designed matching network with the three antennas in HFSS and repeating the simulation, changes in the S11 parameter were observed to see how the impedance had been adjusted. Optimization was performed in case the impedance match was not perfect. Optimization requires changing the circuit components and values in the matching network. This process can be repeated until the impedance is close to the desired match. Once a satisfactory match is achieved, we can verify the entire system performance (antenna plus matching network) by checking parameters like the VSWR, bandwidth, and radiation pattern to ensure the antenna operates effectively in its intended environment.

**Figure 18:**
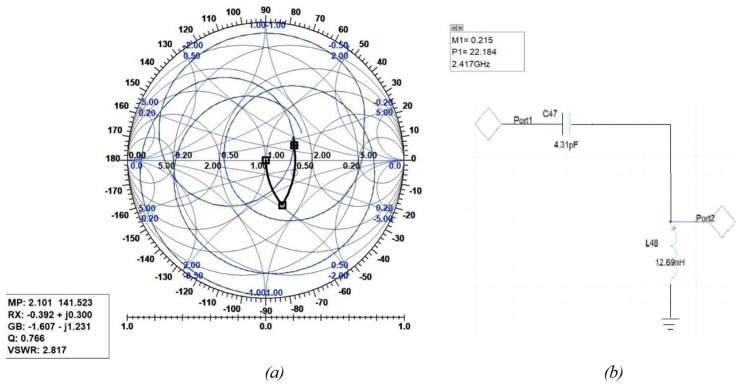
(**a**) Smith Chart for the first assumption of C-shaped antenna. (**b**) Related impedance-matching circuit.

**Figure 19:**
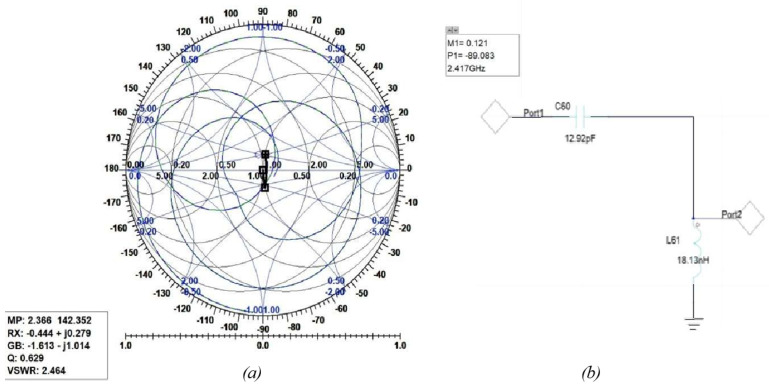
(**a**) Smith Chart for the second assumption of the C-shaped antenna. (**b**) Related impedance-matching circuit.

**Figure 20:**
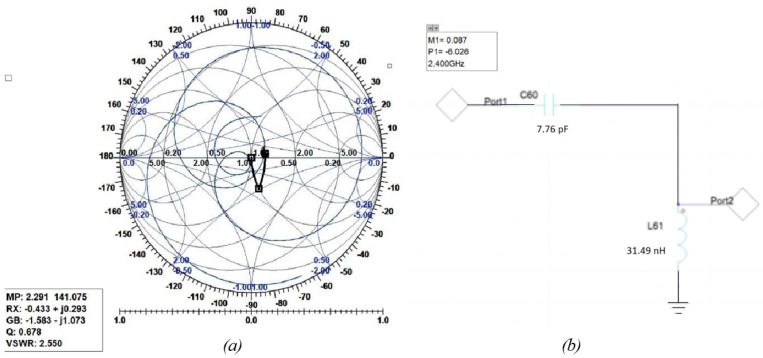
(**a**) Smith Chart for the F-shaped antenna. (**b**) Related impedance-matching circuit.

The following figures show the graphical implementation of impedance matching by the Smith tool and its equivalent LC circuit with values of components toward 2.4 GHz where the trace was found. Figure 18 shows the Smith tool (a) and circuit (b) for the first assumption of the two-C-shape antenna. Figure 19 shows the Smith tool (a) and circuit (b) for the second assumption of the two-C-shape antenna. Finally, Figure 20 shows the F-shaped antenna’s Smith and impedance-matching circuit.

The impact of implementing an equivalent LC circuit will be reflected in the S11 and VSWR parameters; this will also reduce the mismatch of the antennas. Therefore, this process allows an antenna to radiate at the intended frequency with minimal deviation, vastly increasing the performance capabilities.

**Figure 21:**
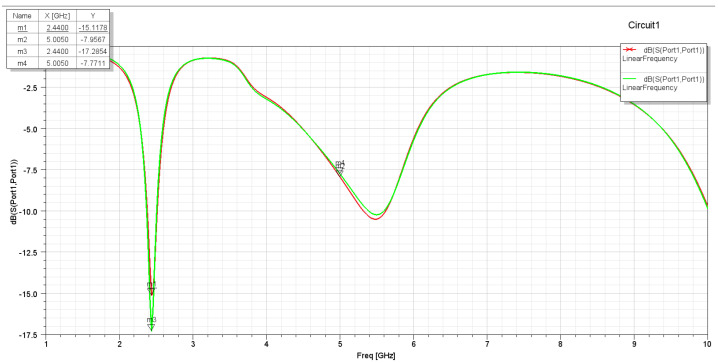
S11 for first assumption two C-Shape antenna after impedance matching network.(The red-colored line represents the S11 for the unmatched antenna, while the green line is for the matched antenna).

**Figure 22:**
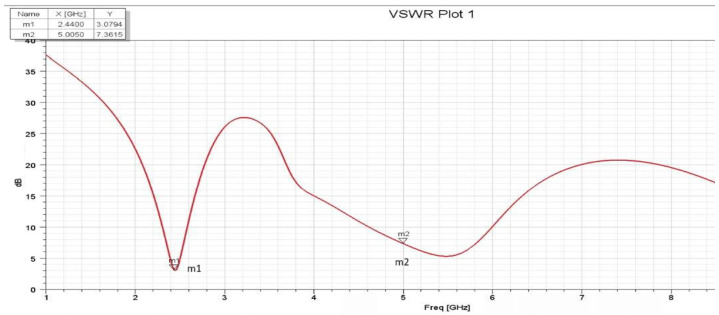
VSWR for first assumption of the two-C-shape antenna after impedance-matching network.

**Figure 23:**
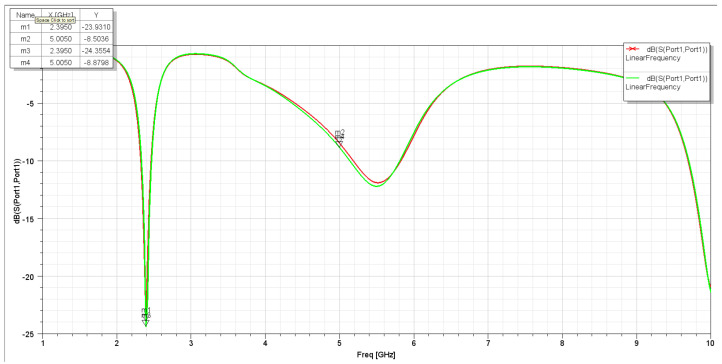
S11 for second assumption of two-C-shape antenna after impedance-matching network.

**Figure 24:**
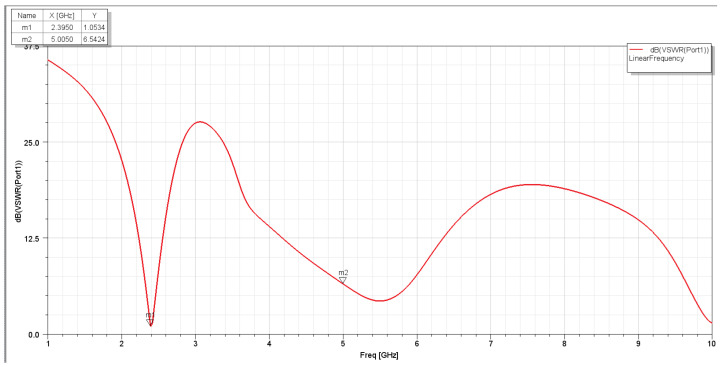
VSWR for second assumption of two-C-shape antenna after impedance-matching network.

Figure 21 and Figure 22 show the S11 and VSWR for the first assumption of the two C-shaped antennas after matching the network, respectively. It is noted that the value in dBs for a matched antenna at 2.4 GHz is lower than without a matching network. And the VSWR is closer to zero at specified frequencies. Similarly, Figure 23 shows the S11 and VSWR for the second assumption of the two C-shaped antennas after matching the network. It is noted that the value in dBs for the matched antenna at 2.4 GHz is almost the same near the 2.4 GHz frequency range. The VSWR before and after applying the impedance-matching circuit is almost the same at certain frequencies (close to zero), as shown in Figure 24.

**Figure 25:**
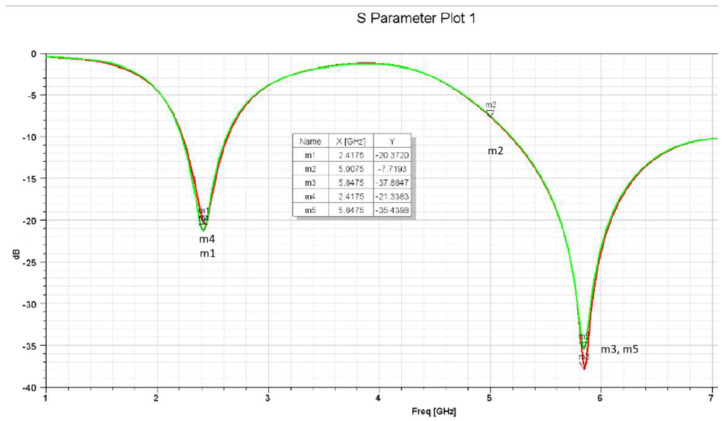
S11 for F-shaped antenna after impedance-matching network.

**Figure 26:**
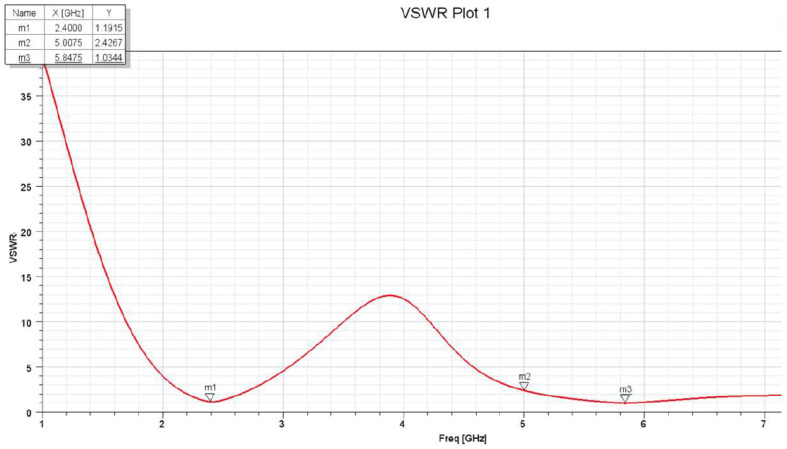
VSWR for F-shaped antenna after impedance-matching network.

The minor changes in the S11 and VSWR values are due to the Smith Chart showing near-real impedance even without applying an impedance-matching circuit. This proves that the design of the antenna is optimized to not only be tuned to around 2.4 and 5 GHz, but also to perfectly match the 50 Ω cable impedance to be connected to its SMA adapter, which means that there is low net reactance, and, hence, the resistance is almost 50 Ω. Figure 25 and Figure 26 show the S11 and VSWR for the F-shaped antenna after matching the network, respectively. It is noted that the value of S11 in dBs for the matched antenna at 2.4 GHz is almost the same. The VSWR after and before impedance matching is almost identical at certain frequencies.

## 5. Conclusions

This research has explored the feasibility and practicality of harvesting RF energy from Wi-Fi frequency bands to power low-power IoT devices. A comprehensive design, simulation, and testing process demonstrated the potential of dual-band antennas operating at 2.4 GHz and 5 GHz for efficient energy capture. Utilizing a high-frequency simulator, we successfully developed and optimized C-shaped and F-shaped microstrip antennas that achieved effective impedance matching and a high RF–DC conversion efficiency. We successfully experimentally tested the proposed C-shaped and F-shaped antennas. According to the simulation and experimental results, the reflection coefficient and VSWR are good enough to harvest enough energy to power IoT devices with suitable impedance matching and high RF–DC conversion stages. The implementation of these antennas in an IoT environment showed promising results. The captured RF energy was converted into usable electrical power that could be directly utilized by IoT devices or stored in energy storage elements for later use. The research addressed the critical need for sustainable energy solutions in IoT applications, particularly in remote locations, where traditional power sources are not viable.

## Figures and Tables

**Table 1:** Design parameters of the proposed antenna.

Parameter	Value mm
W	26
W1	1.8
W2	29
W3	29
L	29
L1	29
L2	29
L3	29
L4	29
L5	26
L6	26
L7	26
L8	26
D1	26
D2	26

**Table 2:** Design parameters of proposed antenna.

Parameter	Value mm
L1	40
L2	28
L3	16.7
L4	34
L5	10
L6	9

**Table 3:** Antenna performance comparison.

Antennas	Two C-Shaped (Assumption I)	Two C-Shaped (Assumption II)	F-Shaped
Geometric size	29 × 26 × 1.6 mm^3^	29 × 26 × 1.6 mm^3^	28 × 40 × 1.6 mm^3^
The dB at 2.4 GHz	−15.11	−23.93	−20.41
The dB at 5 GHz	−7.95	−8.50	−7.62
VSWR at 2.4 GHz	3.07 dB	1.10 dB	1.66 dB
VSWR at 5 GHz	7.36 dB	6.86 dB	7.68 dB
Range of gain in dB	−29.94–5.33	0–3.54	−7.43–2.85

**Table 4:** S11 and VSWR results compared to others’ work.

Antenna Type	Operating Frequency (GHz)	Band Width	S11 Parameter (dB)	VSWR (dB)
[23]	2.4GHz	Dual Band	−1.1 to −2 dB	15.8 dB
[24]	5 GHz	Dual Band	−2 to −2.5 dB	8.7 dB
[25]	2.4 GHz	Dual Band	−2.6 to −3 dB	6.7 dB
[25]	5 GHz	Dual Band	−7.5 to −8 dB	2.5 dB
[26]	2.4 GHz	Ultra-Wide Band	−0.3 to −0.5 dB	57.9 dB
[26]	5 GHz	Ultra-Wide Band	−2.5 to −3 dB	7 dB
[27]	2.4 GHz	Dual Band	−23.8 to −24 dB	1.1 dB
[27]	5 GHz	Dual Band	−8.5 to −9 dB	6.9 dB

**Table 5:** Simulation vs. experimental S11 and VSWR results for two C-shaped and F-shaped antennas at 2.4 GHz.

Antenna Type	S11 Sim	VSWR Sim	S11 Exp	VSWR Exp
Two C-shaped antenna	−23.93 dB	−20.85 dB	1.106 dB	1.2 dB
F-shaped antenna	−20.41 dB	−20.8 dB	1.66 dB	1.2 dB

## Data Availability

The data presented in this study are available on request from the corresponding authors due to their association with simulator requirements.
